# Public Pharmacists' Association

**Published:** 1914-02-07

**Authors:** 


### Public Pharmacists' Association.

The annual general meeting of the Public Pharmacists' and Dispensers' Association was held last week, when Mr. R. W. Lindsey, F.C.S., was in the chair. Mr. J. H. France, Mr. F. N. Clark, Mr. R. Welford, Mr. G. W. Gibson, Mr. A. Howell, Mr. W. H. Windmill, Mr. E. Castles, Mr. J. Cairns, Mr. H. H. Hewitt, Mr. A. H. Jenkin, and others were also present.

The Insurance Committee appointed to deal with matters arising from the National Health Insurance Act had obtained the consent of the National Insurance Commissioners, permitting registered pharmacists acting as dispensers in institutions to join the "Panel of Chemists" and so enable the resident insured staffs to obtain with promptness any medicines, etc., required. The social and educational work of this Association has been maintained vigorously, lectures and demonstrations on radium, *x*-rays, house-flies and disease, London healing wells, and other interesting subjects having been given.

Mr. Lindsey, the retiring chairman, delivered an address, pointing out to the members that persistent hard work was the keynote to success in Association affairs. Brilliant successes cannot always be achieved, but by plodding away some good must eventually fall to the lot of the craft.

The officers appointed for 1914 are as follows: President, Mr. T. H. W. Idris, J.P.; chairman, Mr. J. H. France, M.P.S.; vice-chairman, Mr. R. Welford, M.P.S.; hon. treasurer, Mr. H. H. Hewitt; hon. secretary of Association, Mr. George W. Gibson, M.P.S.; hon. secretary of Poor-Law section, Mr. R. W. Lindsey, F.C.S.; council, Mr. F. N. Clark, Mr. W. Cooper, Mr. A. Jenkin, Mr. A. Howell, Mr. W. E. Kinsman, Mr. W. H. Windmill, Mr. W. E. Miller, and Mr. A. J. Gibbons, these representing pharmacists engaged in Poor Law, general hospitals, mental hospitals, his Majesty's prison service, and provident dispensaries.

All communications concerning membership of the Association should be made to Mr. George W. Gibson, St. Pancras South Infirmary, N.W.

